# Ecophysiological response of native and exotic salt marsh vegetation to waterlogging and salinity: Implications for the effects of sea-level rise

**DOI:** 10.1038/s41598-017-18721-z

**Published:** 2018-02-05

**Authors:** Shi-Hua Li, Zhen-Ming Ge, Li-Na Xie, Wei Chen, Lin Yuan, Dong-Qi Wang, Xiu-Zhen Li, Li-Quan Zhang

**Affiliations:** 1 State Key Laboratory of Estuarine and Coastal Research, Institute of Eco-Chongming, East China Normal University, 200062 Shanghai, China; 2 School of Forest Sciences, University of Eastern Finland, 80101 Joensuu, Finland; 3 Center for Global Change and Ecological Forecasting, East China Normal University, 200062 Shanghai, China; 4 Key Laboratory of Geographic Information Science (Ministry of Education), School of Geographical Sciences, East China Normal University, 200062 Shanghai, China

## Abstract

The ecophysiological characteristics of native *Phragmites australi*s and exotic *Spartina alterniflora* grown under waterlogging and salinity were investigated to explore their adaptation potential to sea level rise. The seasonal course of phenotypic traits, photosynthetic activity and chlorophyll fluorescence parameters of *P. australis* did not change remarkably under shallow flooding, whereas these variables were sensitive to increasing salinity. Waterlogging exacerbated the negative effects of salinity on shoot growth and photosynthetic activity of *P. australis*, and the combined stresses led to an absence of tassel and reproductive organs. By contrast, *S. alterniflora* performed well under both stresses and showed an obvious adaptation of salt secretion with increasing salinity. Light salinity was the optimal condition for *S. alterniflora*, and the tassel growth, chlorophyll content and fluorescence characters under moderate stresses did not differ notably. The Na^+^ and Cl^−^ concentrations in leaves of both species increased, and the K^+^ content decreased in response to salinity. Under moderate and high saline levels, the ion concentrations in *S. alterniflora* were maintained at relatively consistent levels with increased salt secretion. We expect the degradation of *P. australis* and further colonization of *S. alterniflora* under prolonged flooding and saltwater intrusion from sea level rise on the coastline of China.

## Introduction

Coastal salt marshes provide a wide variety of ecological services that include carbon sequestration, pollution reduction, habitat for protected fauna, and shoreline stabilization^1^. However, salt marshes are vulnerable to sea level rise (SLR) because of their low elevation at the intertidal interface^2,3^. Based on the fifth assessment report (AR5) by the Intergovernmental Panel on Climate Change (IPCC^4^), the mean rate of global SLR will be 44–74 cm by 2100. The latest bulletin released by the State Oceanic Administration (SOA) further demonstrated that the rate of SLR in China is higher than the global average^5^. As expected, coastal salt marshes would be at risk of flooding and saltwater intrusion depending on the various SLR projections^6–9^.

Marsh macrophytes play a crucial role in determining community structure, geomorphic processes, primary production, organic matter input, and biogeochemical cycles^10^. Therefore, the response of marsh macrophytes to SLR has been extensively investigated^11–14^. Morris *et al*.^11^ describe a significant correlation between interannual sea level and aboveground biomass for one species in the North Inlet estuary. At low elevation regions with the potential for fast SLR, prolonged waterlogging would lead to reduced vegetation growth and rapid marsh submergence. In the brackish estuary of the Chesapeake Bay, biomass growth of *Spartina americanus* is highest at an optimum elevation, but *Spartina patens* shoot growth decreases at the low intertidal zone with an increase in prolonged waterlogging^12^. Snedden *et al*.^13^ report a strong negative response of *S. patens* biomass to flooding and high salinity in the Breton Sound estuary.

The intrinsic physiological properties of marsh macrophytes in response to environmental stress conditions have been emphasized^14–16^. For instance, the photosynthetic apparatus of *Phragmites australis* is damaged and amino acid and carbohydrate patterns are modified by flooding^17,18^. Salinity stress can impair biosynthesis or accelerate pigment degradation because of increased levels of toxic cations, leading to limited photosynthesis and growth of plants^19^. Although much is known about the general responses of grass species to flooding and salinity, less is known about the variable responses of different marsh species to synergetic stresses^13,20^.

Along China’s coastline, the native *P. australis* is the dominant primary producer and constitutes the community structure in many salt marshes^21^. However, *Spartina alterniflora*, which was introduced to China from North America in the late 1970s, has been expanding rapidly along the Chinese coastline^22,23^. In the Yangtze Estuary, the exotic *S. alterniflora* has a high degree of competitive suppression on many native species (incl. *P. australis*), and ecosystem properties are influenced because of the modification of vegetation structure by *S. alterniflora* invasions^24,25^. Although *P. australis* and *S. alterniflora* are both members of the family Poaceae and have morphological and physiological similarities, *P. australis* is a C_3_ grass, whereas *S. alterniflora* is a C_4_ grass. Therefore, the ecophysiological adaptation of these different marsh macrophytes to environmental stresses could be significantly diverse.

In this study, original mesocosms of *P. australis* and *S. alterniflora* were grown under a waterlogging and salinity gradient, and the ecophysiological responses of the two species throughout an entire growing period were investigated. The primary objective of this study was to determine the effects of single and combined stresses mimicking projected SLR on plant growth, photosynthetic performance, adaptive response (salt secretion), and chlorophyll and ion concentrations in leaves. The species-specific responses of salt marsh C_3_ and C_4_ grasses to waterlogging and salinity stresses will be meaningful in forecasts of the future winner of competition and community structure shifts under conditions of SLR on the Chinese coastline.

## Materials and Methods

### Plant material and experimental setup

Integrated blocks of the plants with soil matrix were excavated from the largest salt marsh of the Chongming Dongtan (121°50′–122°05′E, 31°25′–31°28′N) located on the eastern fringe of Chongming Island in the Yangtze Estuary. In December (winter season) 2015, intact mesocosms consisting of organic soil monoliths (32 cm × 24 cm × 40 cm, a sufficient volume for root growth) with *P. australis* and *S. alterniflora* rhizomes were collected. The sampling sites of the two species were close to one another with similar soil properties, and the plant materials were collected at the same tidal line and had seedlings with similar life forms. Similar volumes of integrated soil monoliths were excavated according to the size of polyethylene containers, and the same soil was used to fill small gaps in the containers.

A total of 48 mesocosms were grown in a ventilated greenhouse with natural light and temperature conditions. A hose with a valve to control drainage was installed on the bottom of each polyethylene container. During January, the mesocosms were watered daily with fresh water to homogenize the soil salinity, which also provided a month of recovery from disturbance before experimental treatments. In early March, the mesocosms were fertilized with revised Hoagland’s nutrient solution^26^ once when the spring buds first appeared. Then, two flooding treatments, non-waterlogging (control group, the water level maintained at half of the container) and waterlogging (the water level maintained 50 mm above the soil surface), and four NaCl-salinity treatments, including freshwater (control group) and 5, 15 and 30 parts per thousand (ppt) salt, were established. The treatments for all containers (2 species × 2 waterlogging treatments × 4 salinity treatments × 3 replicates) were renewed biweekly, and fresh water was used to maintain the water level during the non-irrigation period to avoid excess salt accumulation.

### Measurement of growth characteristics

From March to November, the stem height of three random shoots in each container was measured monthly. Total leaf area of the fully expanded leaves of a single plant was determined *in situ* using a grid paper counting method. In June, August and November, the aerial organs of three plants were harvested, and the biomass was measured. In November (later growing period), the tassels of three plants, with flowers and seeds, were harvested, and the biomass was measured. Harvested leaves, stems and tassels were dried separately in a forced-air oven at 60 °C for constant weight.

### Measurement of photosynthetic parameters

In April, June, September and November, gas exchange measurements were conducted with a 2 cm × 3 cm standard leaf chamber using a portable steady-state photosynthesis system (*Li-6400XT*; Li-Cor Inc., Lincoln, NE, USA). Measurements were performed on the first fully expanded leaves under the flag leaf from 0800 to 1100 h on sunny and generally cloud-free days. Three plants in each container were measured. The net assimilation rates (*A*_N_) at photosynthetic photon flux densities (PPFD) of 0, 20, 40, 60, 80, 100, 200, 400, 600, 800, 1200 and 1600 µmol m^−2^ s^−1^ were measured under a constant 1400 µmol mol^−1^ CO_2_. The light source was a red-blue LED light (*Li-6400-02B*; Li-Cor Inc., Nebraska, USA), and the CO_2_ source for the measurements was the computer-controlled CO_2_ mixing system supplied with the *Li-6400XT* equipment. Leaves were equilibrated at saturating PPFD before initiation of the light response curve. Sufficient time was allowed for the new PPFD to stabilize before logging the measurement (typically requiring 20 min or less). Dark respiration rates (*R*_d_) of the shoots were measured after a period of at least 30 min in the dark. During the measurements, the temperature inside the leaf chamber was maintained at 25 ± 1 °C, and vapor pressure deficit was maintained at approximately 1.0 kPa. The relative humidity of the air in the leaf chamber was set to 60%. The shape of the average light response curve was modeled by fitting the data to a non-rectangular hyperbola equation^27^ using a nonlinear least squares regression. The maximum area-based rate of photosynthesis (*A*_max_) and the apparent quantum yield (*Q*_α_) were used as the indicators of assimilation capacity (see Supplementary information).

### Measurement of chlorophyll fluorescence parameters

In April and October, the chlorophyll fluorescence parameters were determined using an integrated leaf chamber fluorometer (*Li-6400XT-40*; Li-Cor Inc., Lincoln, NE, USA). Following the experimental protocol of Maxwell and Johnson^28^, the maximum (dark-adapted) photochemical efficiency of photosystem II (F_v_/F_m_) and the instant (light-adapted) quantum yield of photosystem II (Φ_PSII_) based on the PPFD-response curves were estimated (see Supplementary information).

Fluorescence was excited with a modulated red radiation of ca. 2 μmol m^−2^ s^−1^ by setting a pulse-width of 3 μs and a frequency of 20 kHz, and a saturating radiation pulse (0.8 s) of ca. 8000 μmol m^−2^ s^−1^ was provided. The minimum chlorophyll fluorescence (F_0_) and the maximal chlorophyll fluorescence (F_m_) of the closed photosystem II center were measured after 30 min of dark-adaptation. Subsequently, the leaves were continuously irradiated, and the fluorescence at the steady state (F_s_) was thereafter recorded; a second saturating pulse ca. 8000 μmol m^−2^ s^−1^ was imposed to determine the maximal fluorescence of the light-adapted state (F_m_′).

### Estimation of salt secretion

During the growing period, salt secretion of *S. alterniflora*, as an adaptive response, was detected under salinity treatments. Because collecting the salt crystalloids secreted at the leaf surface was difficult, leaves were photographed (with scale bar), and the number of salt crystalloids was counted with CorelDRAW Graphics Suite X6. In the graphics interface, ten 0.5 cm × 0.5 cm grids were randomly chosen on each leaf for counting. In September, all the images were collected for both *P. australis* and *S. alterniflora* on the same day from 1500 to 1700 h, when the number of salt crystalloids was generally the highest.

### Measurement of chlorophyll and ion concentrations

Additionally, in September, the leaves undergoing photosynthetic measurements were destructively sampled and washed with deionized water to immediately determine the concentration of chlorophyll. Chl *a* and Chl *b* concentrations were measured according to the methodology described by Inskeep and Bloom^29^. Leaf samples (0.5 g fresh weight) were ground and extracted with 5 mL of 80% acetone, and then the supernatant was measured at 663 and 646 nm with a spectrophotometer (LAMBDA 950 UV/Vis/NIR; PerkinElmer Inc., MA, USA). The ratio of Chl *a*/*b* was also calculated.

Leaf samples were also dried and ground to a fine powder in a ball mill, and then the cations of sodium and potassium (Na^+^ and K^+^) and the anion of chlorine (Cl^−^) were extracted with HNO_3_ and AgNO_3_ standard solutions, respectively. Concentrations of Na^+^ and K^+^ were determined using an atomic absorption spectrophotometer (Schimazu AA 6800; Schimazu Crop, Kyoto, Japan), and the concentration of Cl^−^ was determined with a chloridometer (PXSJ-216; Leica Ltd., SH, China).

### Statistical analyses

All data measured are presented as the mean ± standard deviation (S.D., *n* = 3) throughout the analyses. The results of growth, photosynthesis and chlorophyll fluorescence parameters and leaf characteristics for the native and exotic species were analyzed separately. The significance of the change and single-factor effects of waterlogging and salinity on the variables (except the PPFD-Φ_PSII_ data) were analyzed with one-way analysis of variance (ANOVA) in conjunction with a post hoc Tukey HSD test, and the interactions were tested with two-way ANOVA. The level of statistical significance was set to *P* (probability) <0.05. Relationships between the salt secretion at the leaf surface and ion concentrations in the leaves were evaluated based on the optimal curve-fitting model with the highest coefficient of determination. All analyses of the variables were performed with the SPSS 23.0 statistical software package (*SPSS* Inc., Chicago, IL, USA).

## Results

### Plant growth

**Figure 1:**
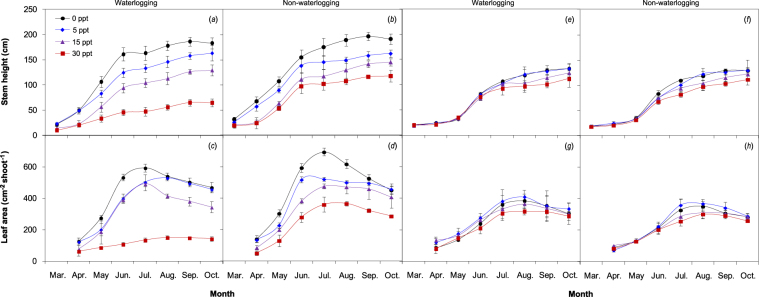
Seasonal variations of stem height and leaf area (mean ± S.D.) of *P. australis* (**a**–**d**) and *S. alterniflora* (**e**–**h**) under waterlogging and salinity treatments.

**Figure 2:**
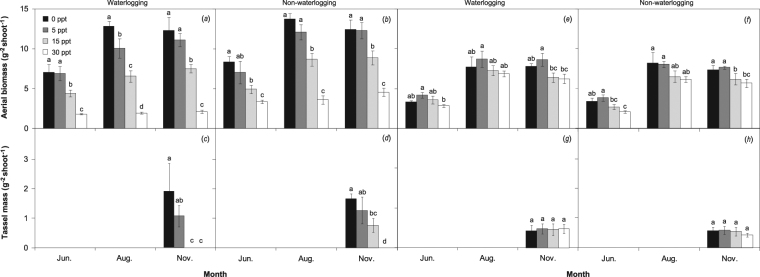
Seasonal variations of aerial biomass and tassel mass (mean ± S.D.) of *P. australis* (**a**–**d**) and *S. alterniflora* (**e**–**h**) under waterlogging and salinity treatments. No tassels (zero value) were recorded under 15 and 30 ppt salinity with waterlogging and 30 ppt salinity under non-waterlogging. Different letters indicate significant differences (*P* < 0.05) among salinity treatments for each measurement period.

Regardless of saline treatment, the stem height and leaf area of *P. australis* under non-waterlogging were on average 15% and 16% higher than those under waterlogging, respectively, whereas in *S. alterniflora*, these differences were marginal throughout the growing period (Fig. 1). The aerial biomass and tassel mass of *P. australis* under non-waterlogging were on average 18% and 24% higher, respectively, than those under waterlogging over the measurement period (November for tassel mass), whereas the difference in *S. alterniflora* was slight regardless of the saline treatment (Fig. 2).

Salinities of 5, 15 and 30 ppt reduced the aerial biomass of *P. australis* by an average of 13%, 42% and 81%, respectively, compared with the control group throughout the measurement period, and the negative response to salinity was more pronounced during the later growing period than in earlier stages. At the end of the growing season, salinity significantly decreased the tassel mass of *P. australis*, and in some cases, tassels did not form under 15 and 30 ppt (Fig. 2). Regardless of flooding treatment, the maximum aerial biomass of *S. alterniflora* was obtained under salinity of 5 ppt, followed by non-salinity, 15 ppt and then 30 ppt.

An ANOVA test indicated a significant effect of waterlogging on the aerial biomass of *P. australis* only in August, but a significant effect of salinity on the stem height and leaf area of *P. australis* over the growing season (Table 1). A significant effect of salinity on aerial biomass and tassel mass of *P. australis* was found over June–November (Table 1), but the effect on *S. alterniflora* was not notable (except in August; Table 2). The combined treatments of waterlogging and high salinity (15 and 30 ppt) resulted in the lowest stem height, leaf area, aerial biomass and tassel mass of both *P. australis* and *S. alterniflora*, whereas no strong interactive effect of waterlogging × salinity was observed for the growth parameters.

**Table 1:** Main and interactive effects (*F*-values) of waterlogging and salinity on the growth and photosynthetic parameters in *P. australis* over the growing period. See the Supplementary Table for complete statistical information.

Parameter	Factor	April	June	August–September	October–November
Stem height	Waterlogging (W)	0.77	1.94	0.56	0.44
	Salinity (S)	5.97*	13.29*	10.21*	134.84**
	W × S	0.35	0.59	0.91	2.82
Leaf area	Waterlogging	0.39	1.04	1.09	0.60
	Salinity	43.22**	12.68*	12.86*	8.01*
	W × S	0.60	0.002	0.53	0.99
Aerial biomass	Waterlogging	/	1.20	9.16*	0.89
	Salinity	/	14.67*	12.45*	12.17*
	W × S	/	0.14	0.05	0.33
Tassel mass	Waterlogging	/	/	/	0.38
	Salinity	/	/	/	18.33*
	W × S	/	/	/	0.45
*A*_max_	Waterlogging	0.76	0.083	1.84	0.25
	Salinity	1.89	3.66	20.99**	9.80*
	W × S	0.057	0.15	0.042	0.27
*Q*_α_	Waterlogging	1.14	1.31	0.006	0.001
	Salinity	6.69	13.69*	12.08*	14.08*
	W × S	0.93	0.67	1.28	0.93
F_0_	Waterlogging	0.63	/	/	0.55
	Salinity	11.99*	/	/	13.99*
	W × S	0.005	/	/	0.30
F_m_	Waterlogging	0.86	/	/	0.83
	Salinity	1.90	/	/	8.25*
	W × S	0.007	/	/	0.05
F_v_/F_m_	Waterlogging	1.97	/	/	0.001
	Salinity	2.05	/	/	4.00
	W × S	0.19	/	/	0.40

*Significance at *P* < 0.05; **significance at *P* < 0.01.

**Table 2:** Main and interactive effects (*F*-values) of waterlogging and salinity on the growth and photosynthetic parameters in *S. alterniflora* over the growing period. See the Supplementary Table for complete statistical information.

Parameter	Factor	April	June	August–September	October–November
Stem height	Waterlogging (W)	0.25	1.18	0.06	0.34
	Salinity (S)	5.33	5.42	61.77*	12.22*
	W × S	0.33	0.12	0.001	0.005
Leaf area	Waterlogging	1.00	1.78	6.65	7.05
	Salinity	0.22	1.29	14.92*	3.31
	W × S	0.006	0.001	0.001	0.21
Aerial biomass	Waterlogging	/	1.68	2.06	0.82
	Salinity	/	6.73	14.21*	4.38
	W × S	/	1.07	1.27	0.08
Tassel mass	Waterlogging	/	/	/	4.74
	Salinity	/	/	/	0.93
	W × S	/	/	/	2.98
*A*_max_	Waterlogging	1.74	2.25	0.034	0.02
	Salinity	5.16	3.75	5.02	4.39
	W × S	0.97	0.07	0.02	0.001
*Q*_α_	Waterlogging	2.99	4.49	2.79	5.37
	Salinity	6.06	6.25	2.95	15.37*
	W × S	0.008	0.09	0.002	0.13
F_0_	Waterlogging	3.27	/	/	2.03
	Salinity	6.13	/	/	9.75*
	W × S	0.03	/	/	1.56
F_m_	Waterlogging	1.93	/	/	5.32
	Salinity	2.32	/	/	5.82
	W × S	0.08	/	/	0.001
F_v_/F_m_	Waterlogging	0.001	/	/	3.90
	Salinity	1.30	/	/	2.73
	W × S	0.006	/	/	0.55

*Significance at *P* < 0.05.

### Photosynthetic efficiency

**Figure 3:**
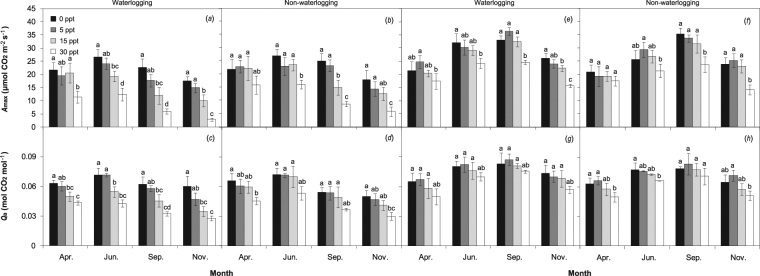
Seasonal variations of the maximum rate of photosynthesis (*A*_max_, mean ± S.D.) and apparent quantum yield (*Q*_α_, mean ± S.D.) of *P. australis* (**a**–**d**) and *S. alterniflora* (**e**–**h**) under waterlogging and salinity treatments. Different letters indicate significant differences (*P* < 0.05) among salinity treatments for each measurement period.

Generally, the *A*_max_ and *Q*_α_ of *P. australis* increased from the early growing period and reached a peak in the summer season (e.g., June) and subsequently declined during the autumn season (Fig. 3). The peak *A*_max_ in *S. alterniflora* was observed in early autumn (September), reflecting a longer growing period than that of *P. australis*. Regardless of saline treatment, the *A*_max_ of *P. australis* under non-waterlogging was on average 12% higher than that under flooding over the measurement period, whereas the differences of *A*_max_ and *Q*_α_ of *S. alterniflora* were negligible. The photosynthetic parameters of *P. australis* were significantly affected by high salinity from summer to the end of the season (Table 1). The *A*_max_ and *Q*_α_ were 11% and 5%, 23% and 16%, and 56% and 35% lower under 5, 15 and 30 ppt, respectively, than those of the control group throughout the measurement period, and the negative response to salinity was more pronounced during the later growing period than in earlier stages.

Regardless of flooding treatment, the saline treatments of 5 and 15 ppt did not change the *A*_max_ and *Q*_α_ of *S. alterniflora* dramatically compared with those of the control group, and 5 ppt salinity generally resulted in the maximum values during summer and autumn. The effect of salinity on the photosynthetic parameters of *S. alterniflora* was not notable (except *Q*_α_ in November; Table 2). The lowest *A*_max_ and *Q*_α_ were measured under the combined treatments of waterlogging and high salinity for *P. australis* and *S. alterniflora*, whereas no significant interactive effect of waterlogging × salinity was detected for the photosynthetic parameters.

### Chlorophyll fluorescence characteristics and chlorophyll content

**Figure 4:**
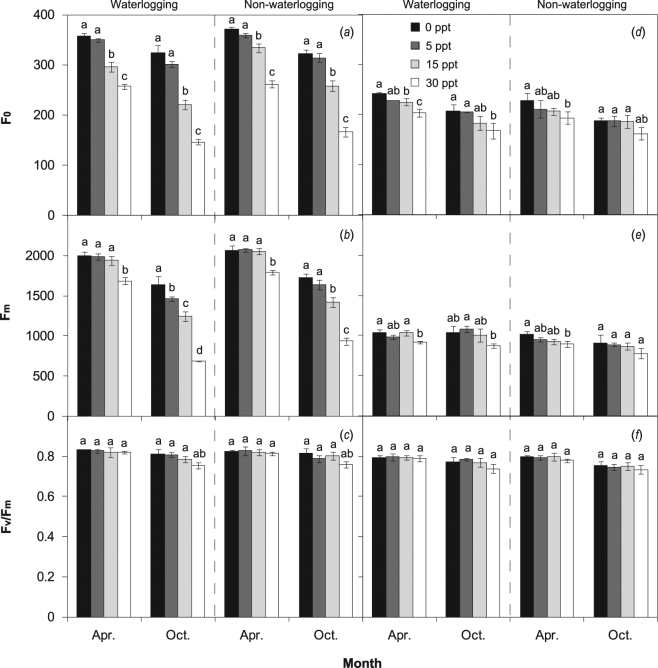
Minimum chlorophyll fluorescence of the open photosystem II center (F_0_, mean ± S.D.), maximal chlorophyll fluorescence of the closed photosystem II center (F_m_, mean ± S.D.), and maximum (dark-adapted) photochemical efficiency of photosystem II (F_v_/F_m_, mean ± S.D.) of *P. australis* (**a**–**c**) and *S. alterniflora* (**d**–**f**) under waterlogging (separated by a dashed line) and salinity treatments in the early and late growing seasons. Different letters indicate significant differences (*P* < 0.05) among salinity treatments for each measurement period.

Regardless of saline treatment, the chlorophyll fluorescence parameters of F_0_ and F_m_ were little affected by flooding treatment in either species at different measurement periods (Fig. 4). With the increase in salinity, the F_0_ and F_m_ of *P. australis* decreased and significantly reduced values were observed under 15 and 30 ppt. An ANOVA test indicated a significant effect of salinity on F_0_ and F_m_ of *P. australis* in both early and late growing periods (Table 1), whereas an effect on F_0_ of *S. alterniflora* only occurred in a later period (Table 2). The lowest values for F_0_ and F_m_ were found for *P. australis* and *S. alterniflora* under the combined treatments of waterlogging and high salinity, whereas the interactive effect of waterlogging × salinity was not significant. For both species, the values of F_v_/F_m_ were slightly altered by waterlogging and saline treatments.

**Figure 5:**
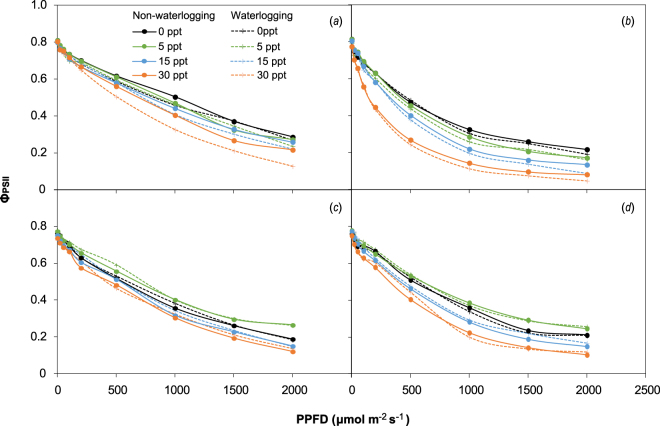
Instant quantum yield (PPFD-adapted) of photosystem II (Φ_PSII_, only means presented) of *P. australis* (**a**,**b**) and *S. alterniflora* (**c**,**d**) under waterlogging and salinity treatments in the early and late growing seasons.

The responses of Φ_PSII_ performance of both species to increasing PPFD showed a linear and then exponential decline to a low level at high PPFD (2000 μmol m^−2^ s^−1^) at earlier and later growing periods, respectively (Fig. 5). Waterlogging slightly affected (except when less than 30 ppt salinity) the Φ_PSII_ of both species at different measurement periods, regardless of the saline treatment. High salinity significantly decreased the Φ_PSII_ of *P. australis* when PPFD was more than 1000 μmol m^−2^ s^−1^ at early growing periods and 500 μmol m^−2^ s^−1^ at the later growing period. Salinity at 5 ppt increased Φ_PSII_ for *S. alterniflora*, whereas high salinity of 15 and 30 ppt slightly decreased values compared with those of the control group. At the later growing period, salinity significantly decreased the Φ_PSII_ of *S. alterniflora* when PPFD was more than 1000 μmol m^−2^ s^−1^.

As measured in early autumn (September), contents of Chl *a* and Chl *b* did not differ significantly between non-waterlogging and waterlogging treatments for both species (Table 3), regardless of saline treatment. Increasing salinity generally reduced the chlorophyll content of *P. australis*, with significantly lower values under salinity of 30 ppt than those in other treatments. However, the effect of salinity on chlorophyll content of *S. alterniflora* was not notable. The waterlogging and saline treatments did not change the ratios of Chl *a*/*b* for either species (with an exception for *S. alterniflora*).

**Table 3:** Concentrations of chlorophyll (mg g^−1^ FW) and cations and anion (μmol g^−1^ DW) in the leaves of *P. australis* and *S. alterniflora* under waterlogging and salinity treatments.

Water level	Salinity	***P. australis***			***S. alterniflora***		
		Chl ***a***	Chl ***b***	Chl a/b ratio	Chl ***a***	Chl ***b***	Chl a/b ratio
Non-waterlogging	0 ppt	1.9 ± 0.1^a^	0.8 ± 0.1^a^	2.5 ± 0.2^a^	1.2 ± 0.1^a^	0.5 ± 0.05^a^	2.5 ± 0.2^a^
	5 ppt	2.0 ± 0.4^a^	0.7 ± 0.1^ab^	2.7 ± 0.5^a^	1.1 ± 0.2^a^	0.5 ± 0.08^a^	2.4 ± 0.3^a^
	15 ppt	1.8 ± 0.2^a^	0.7 ± 0.2^a^	2.3 ± 0.5^a^	1.2 ± 0.2^a^	0.4 ± 0.06^a^	2.6 ± 0.3^a^
	30 ppt	1.6 ± 0.2^b^	0.6 ± 0.1^b^	2.6 ± 0.6^a^	1.2 ± 0.2^a^	0.4 ± 0.05^a^	2.7 ± 0.3^a^
Waterlogging	0 ppt	1.9 ± 0.2^a^	0.7 ± 0.1^ab^	2.5 ± 0.3^a^	1.2 ± 0.1^a^	0.5 ± 0.08^a^	2.4 ± 0.2^ab^
	5 ppt	1.8 ± 0.2^a^	0.8 ± 0.2^a^	2.4 ± 0.4^a^	1.2 ± 0.1^a^	0.5 ± 0.07^a^	2.5 ± 0.2^ab^
	15 ppt	1.8 ± 0.4^a^	0.7 ± 0.2^a^	2.4 ± 0.6^a^	1.3 ± 0.2^a^	0.4 ± 0.09^a^	2.8 ± 0.3^a^
	30 ppt	1.6 ± 0.2^b^	0.6 ± 0.1^b^	2.6 ± 0.4^a^	1.1 ± 0.1^a^	0.5 ± 0.1^a^	2.3 ± 0.2^b^
**Water level**	**Salinity**	***P. australis***			***S. alterniflora***		
		**Na** ^**+**^	Cl^−^	**K** ^**+**^	**Na** ^**+**^	Cl^−^	**K** ^**+**^
Non-waterlogging	0 ppt	46.3 ± 5.8^d^	50.1 ± 4.8^d^	586.4 ± 33.4^a^	155.4 ± 9.4^d^	124.3 ± 8.5^c^	712.5 ± 54.6^a^
	5 ppt	85.4 ± 10.5^c^	74.9 ± 6.3^c^	502.8 ± 49.7^b^	442.0 ± 36.8^c^	251.2 ± 35.3^b^	620.5 ± 38.0^b^
	15 ppt	220.9 ± 18.4^b^	101.8 ± 13.5^b^	459.3 ± 52.5^bc^	910.1 ± 96.5^ab^	576.7 ± 68.8^a^	605.4 ± 74.3^b^
	30 ppt	409.4 ± 52.2^a^	239.6 ± 20.0^a^	315.5 ± 26.8^d^	999.8 ± 68.3^a^	552.3 ± 62.5^a^	584.3 ± 32.5^bc^
Waterlogging	0 ppt	42.6 ± 3.5^d^	42.0 ± 4.6^d^	615.9 ± 49.5^a^	182.5 ± 15.4^d^	149.6 ± 18.6^d^	728.1 ± 49.5^a^
	5 ppt	64.5 ± 8.3^c^	60.7 ± 6.2^c^	495.7 ± 22.8^b^	450.1 ± 29.5^c^	235.6 ± 27.5^c^	604.7 ± 53.7^b^
	15 ppt	203.7 ± 37.0^b^	113.9 ± 9.5^b^	411.8 ± 54.2^c^	933.2 ± 87.6^ab^	575.9 ± 55.0^ab^	577.6 ± 61.2^b^
	30 ppt	382.5 ± 25.6^a^	191.3 ± 21.1^a^	349.2 ± 21.5^d^	1071.4 ± 85.2^a^	627.2 ± 84.3^a^	558.6 ± 33.8^bc^

FW and DW indicate fresh weight and dry weight, respectively. The measurements were conducted in September. Different letters indicate significant differences (*P* < 0.05) among salinity treatments for each variable.

### Salt secretion and ion concentration

**Figure 6:**
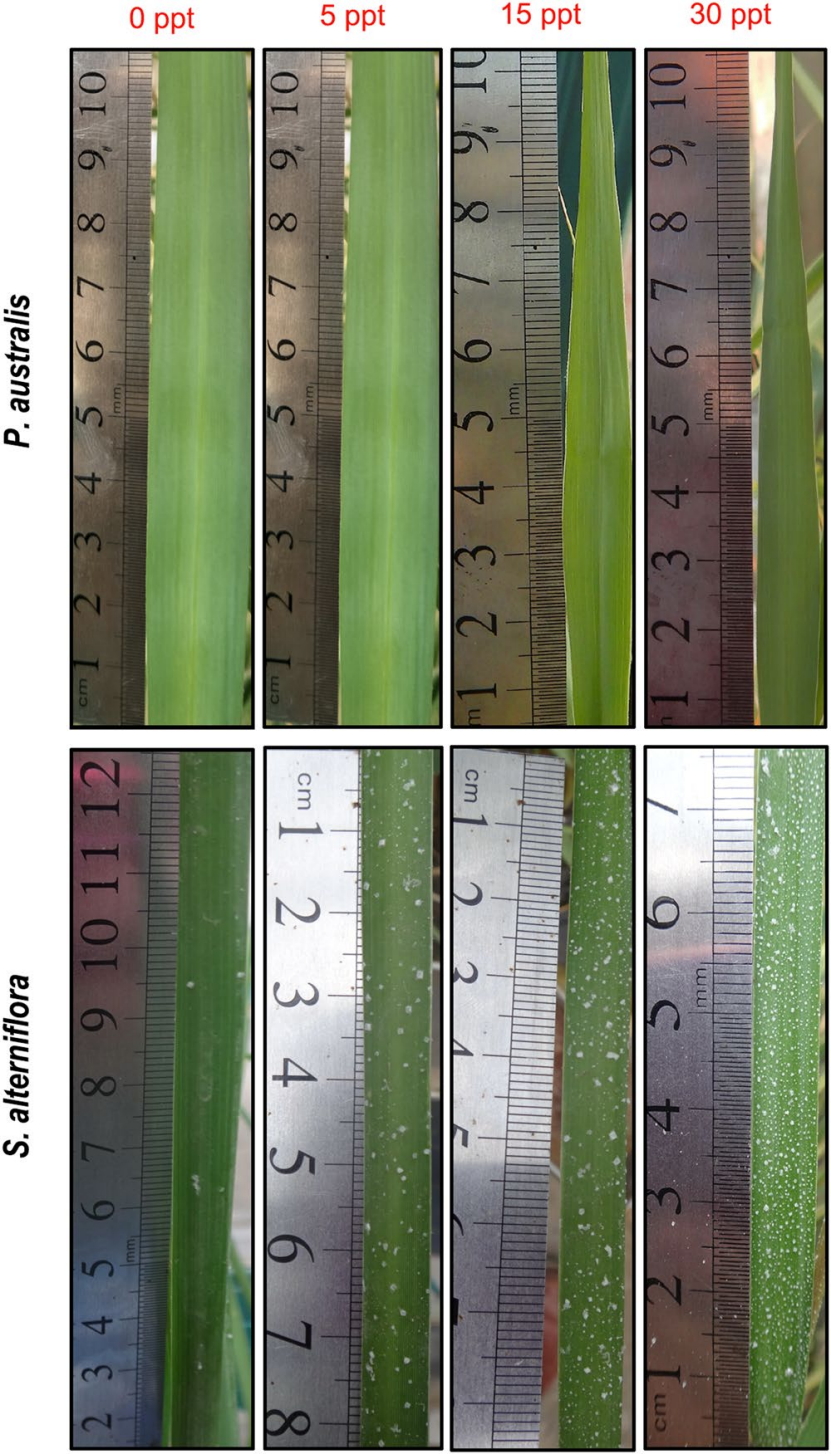
Leaves of *P. australis* (upper panel, no salt secretion) and *S. alterniflora* (bottom panel, distinct salt secretion) subjected to the salinity gradient. The amount of salt secretion was similar between waterlogging treatments. (Photos by S.H.L. and Z.M.G.).

**Figure 7:**
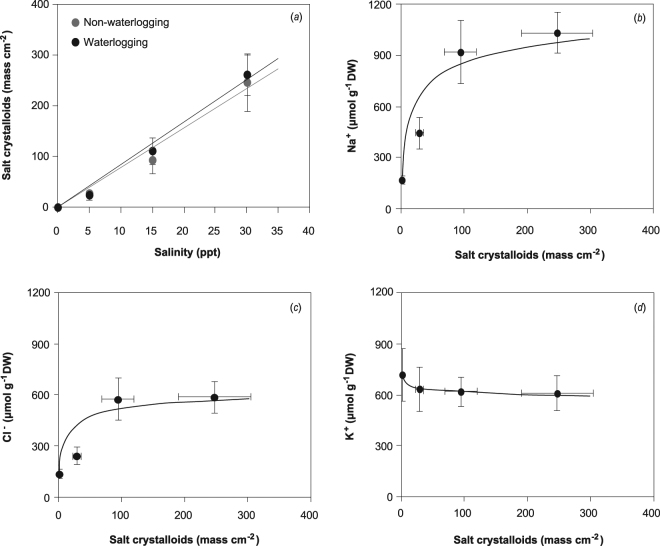
Amount of salt crystalloids secreted on *S. alterniflora* leaf surfaces subjected to the salinity gradient (**a**), and the relationships between salt secretion (on average waterlogging treatments) and Na^+^ concentration (**b**), Cl^−^ concentration (**c**) and K^+^ concentration (**d**) in the leaves.

Over the growing period, *S. alterniflora* secreted salt crystalloids from the leaves and stems, whereas this capacity was absent for *P. australis* (Fig. 6). As measured in September, the amount of salt crystalloids secreted at the leaf surface of *S. alterniflora* increased linearly with increasing salinity (Fig. 7a).

Concentrations of cations and the anion in *P. australis* leaves were higher than those in *S. alterniflora* leaves (Table 3). Regardless of saline treatments, ion concentrations in the leaves of both species under waterlogging did not vary greatly compared with those in non-waterlogging. The concentrations of Na^+^ and Cl^−^ in *P. australis* and *S. alterniflora* leaves increased, whereas the K^+^ content decreased in response to exposure to salinity, with contents significantly different from those of the control. The ion concentrations in *P. australis* leaves differed significantly between each salinity level. The differences of Na^+^ and Cl^−^ concentrations in *S. alterniflora* leaves between the highest two salinity levels (15 and 30 ppt) and the differences of K^+^ concentration among all levels (5, 15 and 30 ppt) were not notable (Table 3).

In *S. alterniflora* leaves, both Na^+^ and Cl^−^ concentrations and salt secretion amount increased, and the K^+^ content decreased with increasing salinity level. When the salinity level was higher than 15 ppt, the ion concentrations remained relatively consistent with the increased amount of salt secretion (Fig. 7b–d).

## Discussion

*P. australis* and *S. alterniflora* are the typical native and exotic species, respectively, in many coastal salt marshes in China^21,22^. In the Yangtze Estuary, the tidal range and the intensity of saltwater intrusion are expected to increase with SLR projections^30^; therefore, plant growth and survival and vegetation structure would be subject to the multiple stresses of prolonged tidal waterlogging and increased salinity.

We found that the seasonal shoot morphology and aerial biomass of *P. australis* were not significantly affected by a single waterlogging treatment, regardless of saline conditions. Additionally, the photosynthetic activity and chlorophyll fluorescence parameters of *P. australis* decreased slightly under waterlogging because of a marginal reduction in photosystem II activity, and the leaf characters of chlorophyll and ion concentrations did not differ significantly. The shallow flooding of 50 mm that was applied in this study provided an explanation for these results. In many coastal salt marshes, *P. australis* primarily occupies high marshes with low tidal water Tables^31–33^. Although grown under a long-term waterlogging (over the entire growing season), the growth and physiological characters of *P. australis* were not influenced remarkably by a low inundation projection. As reported by Mauchamp and Méthy^18^, only a long-term deep submersion resulted in an obvious decline of growth of *P. australis*, caused by severe damage to the photosynthetic apparatus based on fluorescence measurements. Regarding *S. alterniflora*, the aerial biomass was slightly higher under shallow flooding than under non-waterlogging throughout the growing season. Increased biomass with shallow flooding might be linked to the increase in photosynthesis and fluorescence parameters, reflecting that the water-saturated soil matrix might be optimal for the physiological requirements of *S. alterniflora*. In natural marshes, *S. alterniflora* generally colonizes the low marshes with a relatively high water level^33^. Previous studies show that biomass growth of *S. alterniflora* increases with rising sea level up to an optimum flooding frequency, showing greater biomass at intermediate than that at high elevation^11,34^. As reported by Naidoo *et al*.^35^, no substantial metabolic stress response of *S. alterniflora* is observed under waterlogging treatments. We also found that the roots of *S. alterniflora* were much finer and softer than those of *P. australis*, which contain abundant lignin (data not shown); therefore, the soft bulk soil saturated with water might benefit root development and growth of *S. alterniflora*.

However, the shoot development and biomass of *P. australis* were obviously sensitive to an increase in salinity. Therefore, this ecotype of the species in the Yangtze Estuary is salt-sensitive, and saline stress will be of great importance for plant growth and survival in coastal regions. The decreased biomass under saline treatment was primarily attributed to the decline in photosynthetic performance. High salinity suppresses the activity of carboxylation enzymes in chloroplasts and reduces osmotic pressure, resulting in limited photosynthetic activity^36–38^. Under saline conditions, chlorophyll fluorescence parameters (except F_v_/F_m_) of *P. australis* were reduced, indicating a reduction in photochemical efficiency in terms of light absorbed by chlorophyll associated with the photosystem II, reduced regeneration of ribulose-1,5-bisphosphate and the electron translocation rate^39–41^. Our study also showed that the effects of salinity on photosynthetic and fluorescence parameters were more pronounced during the later growing period than in earlier stages, revealing an accelerated degradation of the photosynthetic apparatus with aging. In this study, chlorophyll content of *P. australis* was not notably reduced under salinity of 5 and 15 ppt, which is consistent with previous reports for moderate saline conditions^42^. However, high salinity of 30 ppt significantly reduced the contents of Chl *a* and Chl *b* in *P. australis*. Based on the reviews by Ashraf and Harris^19^ and Sudhir and Murthy^43^, the photosynthetic apparatus of most C_3_ plants is impaired and the chloroplast pigments are degraded under salt stress. In adapting to saline environments, maintenance of intracellular ion homeostasis and osmotic adjustments are essential for plants^44^. The Na^+^ and Cl^−^ contents in *P. australis* leaves under all saline treatments were lower than those in *S. alterniflora* leaves, whereas the rate of increase of ion contents was much greater as the level of salinity increased from 5 to 30 ppt. *Phragmites* spp. generally present downward Na^+^ and Cl^−^ transport between shoot and root and ion accumulation in the shoot tissue in response to salinity^42,45^. Increasing salinity levels significantly decreased the K^+^ concentrations in *P. australis* leaves. Barrett-Lennard and Shabala^46^ suggested that such a change could be attributed to decreases in shoot relative growth rate. Moreover, *P. australis* does not have salt glands in the shoot tissues, and therefore, the capacity for salt secretion is absent, possibly resulting in much more accumulation of excess salt ions and limited uptake of K^+^ in the leaves under high salinity levels.

*S. alterniflora* showed better tolerance to high salinity than that of *P. australis*. Maximal seasonal biomass and photosynthetic parameters in *S. alterniflora* were often observed in the 5 ppt treatment, and the discrepancy of plant growth and photosynthetic activity under 15 ppt was not significantly different from non-salinity. Chlorophyll fluorescence parameters of *S. alterniflora* increased with a small increase in salinity (5 ppt), indicating that the electron transfer and turnover rate of the photosystem II might be promoted. These results are consistent with those of a field investigation of environmental gradients in the Yangtze Estuary showing that *S. alterniflora* grew better than the native species in salt marshes with high salinity^47^. The pattern of phenotypic plasticity of *S. alterniflora* might explain the successful invasion and strong competition with the native species in China^48^. A recent study^49^ found that the growth of *S. alterniflora* under 4 ppt salinity was higher than that in a salt-free environment due to the regulation of antioxidant enzyme activities and the expression of key stress-induced proteins. Those researchers also predicted that *S. alterniflora* might tolerate salinity up to the concentration of local seawater (~12 ppt). *S. alterniflora* also maintained a relatively constant chlorophyll concentration across all salinity levels. The property of C_4_-type photosynthesis of *S. alterniflora* might contribute to some extent to tolerance to salt stress. In the C_4_ pathway, salt ions might facilitate some biochemical processes in photosynthesis and water use efficiency under high salinity^16,50^. The greater salt tolerance of *S. alterniflora* than that of *P. australis* was due to the ability to use Na^+^ for osmotic adjustment in the shoots^45^. Nevertheless, the biomass growth and biochemical parameters of *S. alterniflora* declined significantly under 30 ppt salinity based on the current measurements. Maricle *et al*.^51^ reported that severe salinity decreases the gross rates of O_2_ evolution and net rates of CO_2_ uptake of *S. alterniflora* because of reduced stomatal conductance and increased photoinhibition. Compared with *P. australis*, an obvious adaptive mechanism of *S. alterniflora* to saline environments is salt secretion at the shoot surface with specialized salt glands^52^. With the increase in salinity from 0 to 30 ppt, the amount of salt crystalloids secreted from leaves increased sharply. Although the pattern of change of Na^+^ and Cl^−^ (increase) and K^+^ (decrease) in *S. alterniflora* was the same as that of *P. australis* under saline treatments, the ion concentrations remained relatively consistent under high salinity levels in *S. alterniflora*. This adaptation mechanism is used to adjust osmotic potential and maintain smooth water inflow, in addition to maintaining the balance of K^+^ and Na^+^ in plants, to mitigate the adverse effects of saline stress^53,54^. Recent studies on salt secretion of halophytes indicate that various types of cations and anions can be secreted by salt glands in leaves, but Na^+^ and Cl^−^ are the dominant elements. By comparison, a low-affinity K^+^ transporter inside the plasma membrane of salt glands maintains a high K^+^/Na^+^ ratio in the cell cytosol to mitigate the salt stress^55^.

Although statistical analysis did not show a significant interaction between waterlogging and saline stresses, the synergetic effects on *P. australis* were clear compared with *S. alterniflora*. Even shallow flooding exacerbated the negative effects of salt stress on biomass growth and photosynthetic activity. In particular, plants grown under flooding and 30 ppt salinity had the smallest shoots (stem height and leaf area) and the lowest biomass and photosynthetic capacity compared with those of plants grown under a single stress. The combined hypoxia and salinity are hypothesized to impair the photosynthetic apparatus and disrupt the ion homeostasis between shoots and roots^56,57^. Furthermore, this study showed that the tassel mass with reproductive organs of *P. australis* was significantly reduced under salinity. The combined stresses of waterlogging and salinity also led to an absence of tassel development under 15 and 30 ppt salinity; thus, sexual propagation would clearly be blocked during the next generation. Further monitoring of the developmental response mechanisms (e.g., asexual reproduction by rhizome tissue) and growth under consecutive stresses is required.

Vegetation zonation and spatial patterns are determined by species-specific adaptations to environmental gradients of water level, sediment type and salinity in estuarine marshes^58,59^. With SLR projections, some species would be excluded from the low and middle tidal zones because of weak tolerance to prolonged flooding and higher salinity, and interspecies competition would also lead to a shift of vegetation zonation under environmental stresses^60^. We found that, in addition to phenotypic characteristics, the level of tolerance of photosynthetic performance and biochemical characters to environmental stresses was higher for *S. alterniflora* than that for *P. australis*. Based on these differences, we predict that the native *P. australis* may become more vulnerable to increased flooding and saltwater intrusion, whereas these stresses may favor the exotic *S. alterniflora*. Therefore, our study supports the hypothesis that degradation of native *P. australis* and further colonization of exotic *S. alterniflora* might be driven by a combination of increased flooding and high saltwater loads under projected SLR conditions on the Chinese coastline.

## Electronic supplementary material

Supporting information
