# Supplementary material for: Ecophysiological response of native and exotic salt marsh vegetation to waterlogging and salinity: Implications for the effects of sea-level rise
Source: Sci Rep. 2018 Feb 5;8:2441. doi: 10.1038/s41598-017-18721-z (PMC5799283; doi:10.1038/s41598-017-18721-z)
Supplement: Supplementary file 1 — Supporting information [file 41598_2017_18721_MOESM1_ESM.doc]

**Supplementary information**

**Ecophysiological response of native and exotic salt marsh vegetation to waterlogging and salinity: Implications for the effects of sea-level rise**

Shi-Hua Li^1^, Zhen-Ming Ge^1,2,3^*, Li-Na Xie^1^, Wei Chen^1^, Lin Yuan^1^, Dong-Qi Wang^4^, Xiu-Zhen Li^1^, Li-Quan Zhang^1^

1. *State Key Laboratory of Estuarine and Coastal Research,* *Institute of Eco-Chongming, East China Normal University, 200062 Shanghai, China*
2. *School of Forest Sciences, University of Eastern Finland, 80101 Joensuu, Finland*
3. *Center for Global Change and Ecological Forecasting, East China Normal University, 200062 Shanghai, China*
4. *Key Laboratory for Geographic Information Science (Ministry of Education), School of Geographical Sciences, East China Normal University, 200062 Shanghai, China*

* **Corresponding author**

*E-mail address*: [zmge@sklec.ecnu.edu.cn](mailto:zmge@sklec.ecnu.edu.cn)

Address: Hehai Building, East China Normal University, North Zhongshan Road, 200062 Shanghai, China

Supporting information includes the calculations of photosynthesis and chlorophyll fluorescence parameters and complete statistical information.

**Calculations of photosynthesis and chlorophyll fluorescence parameters**

The shape of the average light response curve was modeled by fitting the data to a non-rectangular hyperbola equation by means of a nonlinear least squares regression.

$A_{\mathrm{N}}=\frac{Q_{\mathrm{a}}\mathrm{PPFD}+A_{\max}-\sqrt{(Q_{\mathrm{a}}\mathrm{PPFD}+A_{\max})^{2}-4\theta Q_{\mathrm{a}}\mathrm{PPFD}A_{\max}}}{2\theta}$ (1)

where *Q*_α_ is the apparent quantum yield (mol mol^−1^, the initial slope of the light response curve), *A*_max_ is the maximum area-based rate of photosynthesis, *θ* is the convexity of the curve.

The maximum (dark-adapted) photochemical efficiency of photosystem II (F_v_/F_m_) and the instant (light-adapted) quantum yield of photosystem II (Φ_PSII_) based on the PPFD-response curves were estimated.

$\mathrm{F}_{\mathrm{v}}/\mathrm{F}_{\mathrm{m}}=\frac{\mathrm{F}_{\mathrm{m}}-\mathrm{F}_{0}}{\mathrm{F}_{\mathrm{m}}}$ (2)

$\Phi_{\mathrm{PSII}}=\frac{\mathrm{F}'_{\mathrm{m}}-\mathrm{F}_{\mathrm{s}}}{\mathrm{F}'_{\mathrm{m}}}$ (3)

where F_m_ is the maximal chlorophyll fluorescence of the closed photosystem II center, F_0_ is the minimum chlorophyll fluorescence of the open photosystem II center, F_m_´ is the maximal fluorescence of light-adapted state and F_s_ is the fluorescence at the steady state.

**Full statistical information**

Supplementary Table 1. Main and interactive effects of waterlogging and salinity on the growth and photosynthetic parameters in *P. australis* over the growing period.

| Parameters | Factors | April | | | June | | | August–September | | | October–November | | |
| --- | --- | --- | --- | --- | --- | --- | --- | --- | --- | --- | --- | --- | --- |
|  |  | *df* | *F* | *P* | *df* | *F* | *P* | *df* | *F* | *P* | *df* | *F* | *P* |
| Stem height | Waterlogging (W) | 1 | 0.77 | 0.43 | 1 | 1.94 | 0.24 | 1 | 0.56 | 0.49 | 1 | 0.44 | 0.54 |
|  | Salinity (S) | 3 | **5.97*** | **0.05** | 3 | **13.29*** | **0.02** | 3 | **10.21*** | **0.03** | 3 | **134.84**** | **0.000** |
|  | W × S | 3 | 0.35 | 0.58 | 3 | 0.59 | 0.48 | 3 | 0.91 | 0.39 | 3 | 2.82 | 0.17 |
| Leaf area | Waterlogging | 1 | 0.39 | 0.56 | 1 | 1.04 | 0.37 | 1 | 1.09 | 0.36 | 1 | 0.60 | 0.48 |
|  | Salinity | 3 | **43.22**** | **0.003** | 3 | **12.68*** | **0.05** | 3 | **12.86*** | **0.05** | 3 | **8.01*** | **0.05** |
|  | W × S | 3 | 0.60 | 0.48 | 3 | 0.002 | 0.96 | 3 | 0.53 | 0.51 | 3 | 0.99 | 0.54 |
| Aerial biomass | Waterlogging | / | / | / | 1 | 1.20 | 0.34 | 1 | **9.16*** | **0.04** | 1 | 0.89 | 0.40 |
|  | Salinity | / | / | / | 3 | **14.67*** | **0.02** | 3 | **12.45*** | **0.02** | 3 | **12.17*** | **0.03** |
|  | W × S | / | / | / | 3 | 0.14 | 0.73 | 3 | 0.05 | 0.84 | 3 | 0.33 | 0.60 |
| Tassel mass | Waterlogging | / | / | / | / | / | / | / | / | / | 1 | 0.38 | 0.57 |
|  | Salinity | / | / | / | / | / | / | / | / | / | 3 | **18.33*** | **0.01** |
|  | W × S | / | / | / | / | / | / | / | / | / | 3 | 0.45 | 0.54 |
| *A*_max_ | Waterlogging | 1 | 0.76 | 0.43 | 1 | 0.083 | 0.79 | 1 | 1.84 | 0.25 | 1 | 0.25 | 0.64 |
|  | Salinity | 3 | 1.89 | 0.24 | 3 | 3.66 | 0.13 | 3 | **20.99**** | **0.01** | 3 | **9.80*** | **0.04** |
|  | W × S | 3 | 0.057 | 0.82 | 3 | 0.15 | 0.72 | 3 | 0.042 | 0.85 | 3 | 0.27 | 0.63 |
| *Q*_α_ | Waterlogging | 1 | 1.14 | 0.35 | 1 | 1.31 | 0.32 | 1 | 0.006 | 0.82 | 1 | 0.011 | 0.92 |
|  | Salinity | 3 | 6.69 | 0.06 | 3 | **13.69*** | **0.02** | 3 | **12.08*** | **0.03** | 3 | **14.08*** | **0.02** |
|  | W × S | 3 | 0.93 | 0.39 | 3 | 0.67 | 0.46 | 3 | 1.28 | 0.32 | 3 | 0.93 | 0.39 |
| F_0_ | Waterlogging | 1 | 0.63 | 0.47 | / | / | / | / | / | / | 1 | 0.55 | 0.50 |
|  | Salinity | 3 | **11.99*** | **0.03** | / | / | / | / | / | / | 3 | **13.99*** | **0.02** |
|  | W × S | 3 | 0.005 | 0.84 | / | / | / | / | / | / | 3 | 0.30 | 0.61 |
| F_m_ | Waterlogging | 1 | 0.86 | 0.41 | / | / | / | / | / | / | 1 | 0.83 | 0.42 |
|  | Salinity | 3 | 1.90 | 0.24 | / | / | / | / | / | / | 3 | **8.25*** | **0.05** |
|  | W × S | 3 | 0.007 | 0.94 | / | / | / | / | / | / | 3 | 0.05 | 0.84 |
| F_v_/F_m_ | Waterlogging | 1 | 1.97 | 0.23 | / | / | / | / | / | / | 1 | 0.001 | 0.97 |
|  | Salinity | 3 | 2.05 | 0.11 | / | / | / | / | / | / | 3 | 4.00 | 0.12 |
|  | W × S | 3 | 0.19 | 0.68 | / | / | / | / | / | / | 3 | 0.40 | 0.56 |

*: significance at *P* < 0.05, **: significance at *P* < 0.01

Supplementary Table 2. Main and interactive effects of waterlogging and salinity on the growth and photosynthetic parameters in *S. alterniflora* over the growing period.

| Parameters | Factors | April | | | June | | | August–September | | | October–November | | |
| --- | --- | --- | --- | --- | --- | --- | --- | --- | --- | --- | --- | --- | --- |
|  |  | *df* | *F* | *P* | *df* | *F* | *P* | *df* | *F* | *P* | *df* | *F* | *P* |
| Stem height | Waterlogging (W) | 1 | 0.25 | 0.88 | 1 | 1.18 | 0.34 | 1 | 0.06 | 0.94 | 1 | 0.34 | 0.59 |
|  | Salinity (S) | 3 | 5.33 | 0.08 | 3 | 5.42 | 0.08 | 3 | **61.77**** | **0.001** | 3 | **12.22*** | **0.03** |
|  | W × S | 3 | 0.33 | 0.60 | 3 | 0.12 | 0.75 | 3 | 0.001 | 0.99 | 3 | 0.005 | 0.83 |
| Leaf area | Waterlogging | 1 | 1.01 | 0.37 | 1 | 1.78 | 0.09 | 1 | 6.65 | 0.06 | 1 | 7.05 | 0.06 |
|  | Salinity | 3 | 0.22 | 0.66 | 3 | 1.29 | 0.32 | 3 | **14.92*** | **0.02** | 3 | 3.31 | 0.14 |
|  | W × S | 3 | 0.06 | 0.82 | 3 | 0.001 | 0.91 | 3 | 0.001 | 0.97 | 3 | 0.21 | 0.67 |
| Aerial biomass | Waterlogging | / | / | / | 1 | 1.68 | 0.26 | 1 | 2.06 | 0.36 | 1 | 0.82 | 0.42 |
|  | Salinity | / | / | / | 3 | 6.73 | 0.06 | 3 | **14.21*** | **0.02** | 3 | 4.38 | 0.10 |
|  | W × S | / | / | / | 3 | 1.07 | 0.36 | 3 | 1.27 | 0.70 | 3 | 0.08 | 0.79 |
| Tassel mass | Waterlogging | / | / | / | / | / | / | / | / | / | 1 | 4.74 | 0.09 |
|  | Salinity | / | / | / | / | / | / | / | / | / | 3 | 0.93 | 0.39 |
|  | W × S | / | / | / | / | / | / | / | / | / | 3 | 2.98 | 0.16 |
| *A*_max_ | Waterlogging | 1 | 1.74 | 0.26 | 1 | 2.25 | 0.21 | 1 | 0.034 | 0.16 | 1 | 0.02 | 0.89 |
|  | Salinity | 3 | 5.16 | 0.09 | 3 | 3.75 | 0.13 | 3 | 5.02 | 0.07 | 3 | 4.39 | 0.10 |
|  | W × S | 3 | 0.97 | 0.38 | 3 | 0.07 | 0.81 | 3 | 0.02 | 0.93 | 3 | 0.001 | 0.98 |
| *Q*_α_ | Waterlogging | 1 | 2.99 | 0.16 | 1 | 4.49 | 0.10 | 1 | 2.79 | 0.17 | 1 | 5.37 | 0.08 |
|  | Salinity | 3 | 6.06 | 0.07 | 3 | 6.25 | 0.06 | 3 | 2.95 | 0.16 | 3 | **15.37*** | **0.02** |
|  | W × S | 3 | 0.008 | 0.93 | 3 | 0.09 | 0.78 | 3 | 0.002 | 0.97 | 3 | 0.13 | 0.74 |
| F_0_ | Waterlogging | 1 | 3.27 | 0.15 | / | / | / | / | / | / | 1 | 2.03 | 0.23 |
|  | Salinity | 3 | 6.13 | 0.07 | / | / | / | / | / | / | 3 | **9.75*** | **0.04** |
|  | W × S | 3 | 0.03 | 0.96 | / | / | / | / | / | / | 3 | 1.56 | 0.28 |
| F_m_ | Waterlogging | 1 | 1.93 | 0.24 | / | / | / | / | / | / | 1 | 5.32 | 0.08 |
|  | Salinity | 3 | 2.32 | 0.20 | / | / | / | / | / | / | 3 | 5.82 | 0.07 |
|  | W × S | 3 | 0.08 | 0.93 | / | / | / | / | / | / | 3 | 0.001 | 0.98 |
| F_v_/F_m_ | Waterlogging | 1 | 0.001 | 0.97 | / | / | / | / | / | / | 1 | 3.90 | 0.12 |
|  | Salinity | 3 | 1.30 | 0.32 | / | / | / | / | / | / | 3 | 2.73 | 0.17 |
|  | W × S | 3 | 0.006 | 0.82 | / | / | / | / | / | / | 3 | 0.55 | 0.49 |

*: significance at *P* < 0.05
